# Angiotensin (1–7) Improves Pancreatic Islet Function via Upregulating PDX-1 and GCK: A Dose-Dependent Study in Mice

**DOI:** 10.1155/ije/1672096

**Published:** 2024-12-19

**Authors:** Ziwei Lin, Jiaqi Lin, Anqi Huang, Zixu Zhang, Xinyi Wu, Guoshu Yin, Chiju Wei, Wencan Xu

**Affiliations:** ^1^Shantou University Medical College, Shantou, China; ^2^Department of Endocrinology and Metabolism, The First Affiliated Hospital of Shantou University Medical College, Shantou, China; ^3^Multidisciplinary Research Center, Shantou University, Shantou, China

**Keywords:** angiotensin (1–7), diabetes mellitus, GCK, islet cells, PDX-1

## Abstract

**Purpose:** This study aimed to verify the effect of angiotensin (1–7) on improving islet function and further explore the signaling pathway that may be involved in this improvement. It also aimed to explore the effects of angiotensin (1–7) on blood glucose levels, islet function, and morphological changes in db/db mice and its potential signal pathway.

**Methods:** Forty-five db/db mice were divided randomly into a model control group and different doses of angiotensin (1–7) intervention groups (0, 150, 300, and 600 *μ*g/kg/d), while seven db/m mice were assigned as the normal control group. The angiotensin (1–7) intervention groups received daily intraperitoneal administration for 8 weeks, whereas the normal control group was injected intraperitoneally with an equal volume of normal saline every day for 8 weeks. Changes in weight and food intake of mice were detected. Effect of angiotensin (1–7) on lipid metabolism, islet function, the morphology of pancreatic islets, and *β*-cell mass on mice were evaluated. The expression of PDX-1 and GCK in pancreatic tissue was verified.

**Results:** The group receiving angiotensin (1–7) at a dosage of 600 *μ*g/kg/d showed a significant decrease in body weight, triglyceride levels, and fasting blood glucose, along with an improvement in glucose tolerance. In the 300 *μ*g/kg/d group, angiotensin (1–7) tended to increase the total volume of islets. Moreover, the intervention groups exhibited a significant increase in the ratio of *β* cells, small islets (30–80 *μ*m in diameter), as well as the expression levels of PDX-1 and GCK in pancreatic tissue.

**Conclusion:** Angiotensin (1–7) could improve glucose and lipid metabolism and islet function by promoting the expression of PDX-1 and GCK genes in the pancreas of db/db mice.

**Summary**

• Angiotensin (1–7) improved glucose and lipid metabolism in db/db mice.
• Angiotensin (1–7) increased the total volume and number of islets as well as the beta-cell ratio and beta-cell mass in db/db mice.
• Angiotensin (1–7) activated the expression of PDX-1 and GCK in the pancreas of db/db mice.

## 1. Introduction

In recent decades, with the rapid development of the economy, changes in lifestyle, and the aging of population, the incidence rate and prevalence of type 2 diabetes mellitus (T2DM) in the world have increased dramatically, especially in obese people. Hyperinsulinemia often accompanies abnormal lipid levels, and insulin resistance is considered as the underlying cause of these characteristics, leading to high cardiovascular morbidity and mortality [1–3]. The classical renin–angiotensin system (RAS) plays a crucial role in regulating cardiovascular function, blood pressure, neuroendocrine balance, and electrolyte homeostasis. Both exocrine and endocrine cells in pancreatic tissue express components of the RAS [4]. The pancreatic RAS is influenced by various physiological and pathophysiological factors, which have significant effects on the structure and function of the pancreas. Angiotensin II (Ang II), the primary active peptide of the RAS, exerts vasoconstriction, oxidative stress, fibrosis, and inflammatory responses by binding to the angiotensin type 1 receptor (AT1R). Ang II exacerbates vascular endothelial injury, impairs blood flow perfusion to pancreatic islets, reduces blood flow within the islets, and subsequently impairs glucose-stimulated first-phase insulin secretion [5]. Numerous studies have demonstrated a close association between RAS activation and metabolic syndrome, highlighting its pivotal role in the development and progression of diabetes.

In addition to the classic ACE-AngII-AT1 receptor axis in the RAS, a newly discovered ACE2-Ang(1–7)-Mas receptor axis has been found to play a significant role in the pathogenesis of diabetes. Angiotensin (1–7) is an active 7-peptide composed of aspartic acid, histidine, valine, proline, arginine, and tyrosine. It is primarily generated through ACE2 hydrolysis of Ang II and Ang I and exerts its effects by activating the G-protein coupled receptor Mas [6, 7]. Studies have shown that mice with a specific knockout of the Mas gene exhibit insulin resistance and metabolic disorders related to glycolipids [8]. Further exploration of the RAS has revealed that ACE2, Ang-(1–7), and Mas constitute the ACE2-Ang-(1–7)-MAS pathway, which produces biological effects that oppose the activation of AT1R. Pancreatic duodenal homeobox factor-1 (PDX-1), also known as somatostatin transcription factor (stf-1), belongs to the homeobox family and serves as a key transcription factor for the insulin gene in *β* cells. It plays a crucial role in maintaining normal pancreatic islet function and *β*-cell activity [9]. Glucokinase (GCK), also referred to as hexokinase IV, is an enzyme involved in the initial step of glucose metabolism. It catalyzes the conversion of glucose to glucose-6-phosphate. Acting as a glucose receptor, GCK regulates insulin release and plays a vital role in maintaining blood glucose homeostasis [10]. However, even though a large number of studies have confirmed the effects of angiotensin (1–7) on the RAS system and *β* cells, limited research has been conducted on the impact of angiotensin (1–7) on islet function. Furthermore, the underlying mechanism by which angiotensin (1–7) plays a key role in reducing glucose levels and improving islet function remains unclear.

This study aimed to investigate the effects of angiotensin (1–7) on blood glucose levels, islet function, and morphological changes in db/db mice. To achieve this, three different doses of angiotensin (1–7) were administered. The expression of PDX-1 and GCK was detected to explore the potential signal pathway involved in improving islet function. The goal of our research is to shed light on the impact of angiotensin (1–7) and identify the underlying signaling mechanisms that may contribute to these changes. Ultimately, these findings will provide valuable insights for the treatment of diabetes, offering new avenues for therapeutic interventions.

## 2. Methods

### 2.1. Animal Feeding and Intervention Stage

Forty-five 6-week-old specific-pathogen-free (SPF) grade male db/db mice were obtained from Jiangsu GemPharmatech, while seven 7-week-old SPF grade male db/m mice were obtained from Changzhou Cavens. After a 3-week adaptation period, the db/db mice were randomly divided into four groups: Control, Ang-(1–7) 150, Ang-(1–7) 300, and Ang-(1–7) 600. These groups represented intraperitoneal injection doses of 0, 150, 300, and 600 *μ*g/kg/d of Ang-(1–7), respectively. The db/m mice served as the normal control group (NC group). Throughout the experiment, all db/db mice had free access to water and high-fat diet (HFD). The HFD-treated groups were fed ad libitum with a 60 kcal% HFD obtained from Research Diets (D12495). On the other hand, the mice from the NC group were fed a regular diet with 10 kcal% fat content (HFK Bioscience, H10010) and regular water. Each intervention group and the model control group consisted of 11 and 12 db/db mice, respectively, while the normal control group comprised 7 db/m mice. The normal control group received an intraperitoneal injection of an equivalent volume of normal saline.

The mice were weighed on a weekly basis for a duration of 12 weeks, and their food intake was measured in cages each week. Fasting blood glucose (FBG) was monitored, and an intraperitoneal glucose tolerance test (IPGTT) was conducted during the ninth week. On the 11th week, an intraperitoneal insulin tolerance test (IPITT) was administered. All experimental protocols and procedures were thoroughly reviewed and approved by the Ethics Committee of Experimental Animals of Shantou University (SUMC2020-328).

### 2.2. Serum and Tissue Collection

Prior to sacrifice, the mice were subjected to anesthesia by intraperitoneal injection of 2% pentobarbital sodium at a dose of 50 mg/kg. This procedure was conducted after a 12-h fasting period with unrestricted access to water the night before. Blood samples were collected via eyeball extraction, and the supernatant was obtained by centrifugation. The collected samples were then stored at −80°C for further analysis. Following the collection of blood samples, the liver and pancreas tissues of the mice were carefully dissected and fixed in 4% paraformaldehyde (PFA) solution. The fixed tissues were subsequently processed for paraffin sectioning, which allowed for the preparation of thin tissue slices suitable for microscopic examination and analysis.

### 2.3. Serum Biochemical and Insulin Parameters

Serum triglycerides (TGs), total cholesterol (TC), high-density lipoprotein (HDL), low-density lipoprotein (LDL), and glucose were measured using an automated Unicel DXC 800 Chemistry Analyzer. Serum insulin was measured by enzyme-linked immunosorbent assay (ELISA) (CEA448Mu, CLOUD-CLONE CORP., Wuhan, China).

### 2.4. Glucose Tolerance Test (GTT) and Insulin Tolerance Test (ITT)

IPGTT was carried out during the ninth week, while IPITT was conducted in the 11th week. To perform these tests, a 1–2-mm section of the tail tip of each mouse was cut. The first drop of blood was discarded, and the second drop was used for blood glucose measurement using a Sannuo glucometer (Sannuo, China). In the ninth week, the mice were fasted overnight for 6 h and were injected intraperitoneally with glucose at the amount of 1 g/kg of the IPGTT. The blood glucose levels were measured and recorded at 0, 15, 30, 60, and 120 min, respectively. In the 11th week, the mice were fasted overnight for 6 h and were injected intraperitoneally according to the insulin content of 1 U/kg (Novolin R, Novo Nordisk company, Denmark) of the IPITT. The blood glucose levels were measured and recorded at 0, 15, 30, 60, and 120 min, respectively.

### 2.5. Determination of Islet Number and Size and Hematoxylin–Eosin (HE) Staining

The number and size of the islets were evaluated by HE staining as previously reported [11]. All sections of pancreatic tissue and liver tissue blocks were subjected to HE staining. Five mice were randomly selected from each group. Thirty pieces of pancreatic tissue wax blocks of mice from each group (5 *μ*m thickness serial sections with 100 *μ*m intervals between each slice) were used to scan and photograph all islets using Image-Pro Plus8.0 software to calculate the number and size of islets. The sum of the number of islets under the pieces is the number of islets in the group. The median of the shortest and longest diameters of the islets was the size of the islets.

### 2.6. Immunofluorescence Staining

Sections were incubated with Rabbit Anti-Insulin (1:200, ab181547, Abcam) overnight at 4°C and then incubated with goat serum (diluted 1:500, ab150077, Abcam) the next day. Five tissue wax blocks were randomly selected from each group. Under the same magnification, the islets of each slice were photographed in a panoramic view (80 islets were selected in each group). The proportion of *β* cells was analyzed under Image-Pro Plus8.0 software.

### 2.7. Quantitative Real-Time PCR Analysis

Total RNA was extracted from ground tissue. The concentration and purity of RNA were measured using ultraviolet spectrophotometer measurements. PrimeScript RT Reagent Kit (TaKaRa, Japan) was used to remove gDNA from RNA samples and reverse transcribe RNA into cDNA. Primers (BGI) were: GCK-forward: AGGAGGCCAGTGTAAAGATGT, GCK-reverse: CTCCCAGGTCTAAGGAGAGAAA; PDX-1-Forward: GAGGTGCTTACACAGCGGA, PDX-1-Reverse: GGGGCCGGGAGATGTATTT; *β*-actin-Forward: ACGGCCAGGTCATCACTATTG, *β*-actin-Reverse: CAAGAAGGAAGGCTGGAAAAGA. The qPCR Kit (Takara, Japan) was used to specifically amplify using cDNA as a template; 95°C pre-denaturation for 5 min, 94°C denaturation for 30 s, 55°C annealing for 40 s, 72°C extension for 30 s, 28–36 cycles, 72°C extension for 7 min, storage at 4°C. Finally, the relative expression level of the target gene in each group was calculated.

### 2.8. Immunohistochemistry (IHC)

The sections to be tested were deparaffinized, hydrated, antigen retrieved, blocked using an IHC Kit (MXB), and incubated with primary anti-GCK antibody (OTI3E3, Novus) and anti-PDX-1 antibody (ab219207, Abcam) overnight. Negative control was added dropwise to PBS solution and rewarmed the next day. The secondary antibody was incubated for 60 min and washed with PBS for 3 min per wash. DAB staining solution kit (KIT-5920, MXB) was used for the color reaction. Once the desired staining intensity was achieved, the reaction was stopped, and the nuclei were counterstained with hematoxylin. Following this, the sections were rinsed with running water for 3 min, dehydrated using a gradient alcohol solution, and sealed with neutral resin. Microscopic images of the stained sections were captured, and semiquantitative analysis of the staining was conducted using Image-Pro Plus 8.0 software.

### 2.9. Western Blot Analysis

Tissue samples were ground after adjusting the lysate-to-tissue weight ratio to 6:1. The supernatant was collected by centrifugation. Protein concentration in the supernatant was determined using the BCA kit (P0010S, Beyotime, the Netherlands). Polyacrylamide gels (P0903S, Beyotime, the Netherlands) were prepared for protein gel electrophoresis. Subsequently, the separated proteins were transferred onto PVDF membranes. After washing the membrane with TBST, it was blocked for 90 min (5% skim milk) with anti-PDX-1 antibody (ab219207; Abcam) and anti-GCK antibody (ab184169; Abcam) and incubated overnight at room temperature on a shaker. The membrane was washed with TBST solution 3 times and then incubated with horseradish peroxidase coupled secondary antibody (#91196, CST) for 1 h. The protein bands were detected by chemiluminescence (ChemiDoc xr-s, Bio-Rad, USA). For normalization, the same membrane was immunoblotted with anti-*β*-actin antibody (ab8226; Abcam).

### 2.10. Statistical Analyses

The results were shown as mean ± standard deviation. All statistical analyses were performed by one-way ANOVA in GraphPad Prism 8.0 software. Prior to parametric analysis, the normality of the dependent variable was assessed by Shapiro–Wilk's normality test. The homogeneity of variances was assessed by Levene's test. Values of *p* < 0.05 were considered statistically significant.

## 3. Results

### 3.1. Angiotensin (1–7) Decreased Weight in Mice

**Figure 1:**
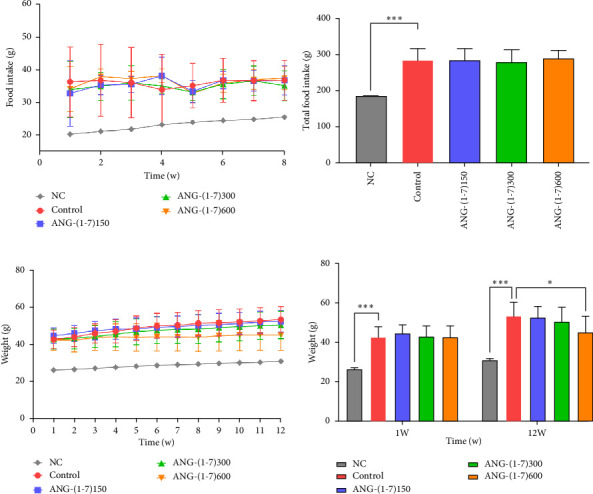
Changes in weight and food intake of mice. (a) Line chart of food intake; (b) histogram total food intake; (c) line chart of body weight; (d) histogram of body weight. Bars represent the mean ± SD. Compared to control: ⁣^∗^*p* < 0.05, ⁣^∗∗∗^*p* < 0.001. *n* = 7–11 for each group.

The angiotensin (1–7) intervention group and Control group had similar food intake, displaying no significant difference (Figures 1(a) and 1(b)). Prior to the intervention, both groups exhibited similar body weights, without any statistically significant distinction (Figure 1(c)). As the intervention time progressed, the body weight in the Ang-(1–7) intervention group decreased compared with the control group. After 12 weeks of intervention, the average weights for the groups of NC, control, ANG-(1–7)150, ANG-(1–7)300, and ANG-(1–7)600 were 31.09, 53.32, 52.45, 50.36, and 45.08 g, respectively. Notably, the body weight of the ANG-(1–7)600 group exhibited a significant decrease (Figure 1(d)).

### 3.2. Angiotensin (1–7) Improved Lipid Metabolism in db/db Mice

**Figure 2:**
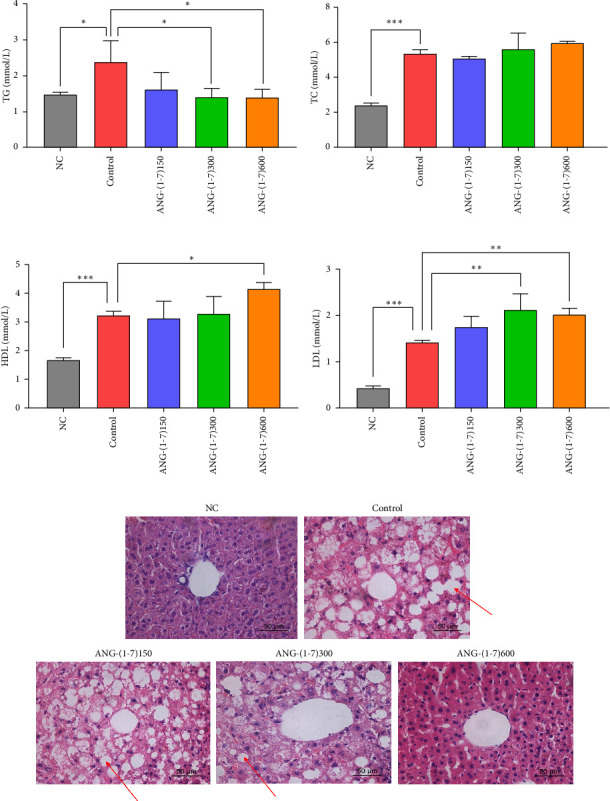
Effect of angiotensin (1–7) on lipid metabolism in db/db mice. (a) Serum triglycerides (TG) in different groups after intervention. (b) Serum total cholesterol (TC) in different groups after intervention. (c) Serum high-density lipoprotein (HDL) in different groups after intervention. (d) Serum low-density lipoprotein (LDL) in different groups after intervention. Each bar represents the mean ± SD. Compared to control: ⁣^∗^*p* < 0.05, ⁣^∗∗^*p* < 0.01, ⁣^∗∗∗^*p* < 0.001. *n* = 4 for each group. (e) Representative image of liver tissue stained with HE from NC group, control group, ANG-(1–7)150 group, ANG-(1–7)300 group, and ANG-(1–7)600 group. Original magnification ×400.

Compared with the control group, serum TGs in the angiotensin (1–7) intervention group decreased to various degrees (Figure 2(a)). The average of TG in the angiotensin (1–7) intervention group was in the range of 1.41–1.46 mmol/L compared to 2.39 mmol/L in the control group. However, there was no significant difference observed in TC levels within the angiotensin (1–7) intervention groups (Figure 2(b)). Furthermore, the serum levels of HDL and LDL displayed an upward trend (Figures 2(c) and 2(d)), with statistically significant differences detected. Notably, histological examination of liver tissues using HE staining demonstrated a reduction in liver fat content in the angiotensin (1–7) intervention group. Additionally, hepatocytes appeared uniform in size and exhibited normal arrangement (Figure 2(e)).

### 3.3. Angiotensin (1–7) Improved FBG and Glucose Tolerance in db/db Mice

**Figure 3:**
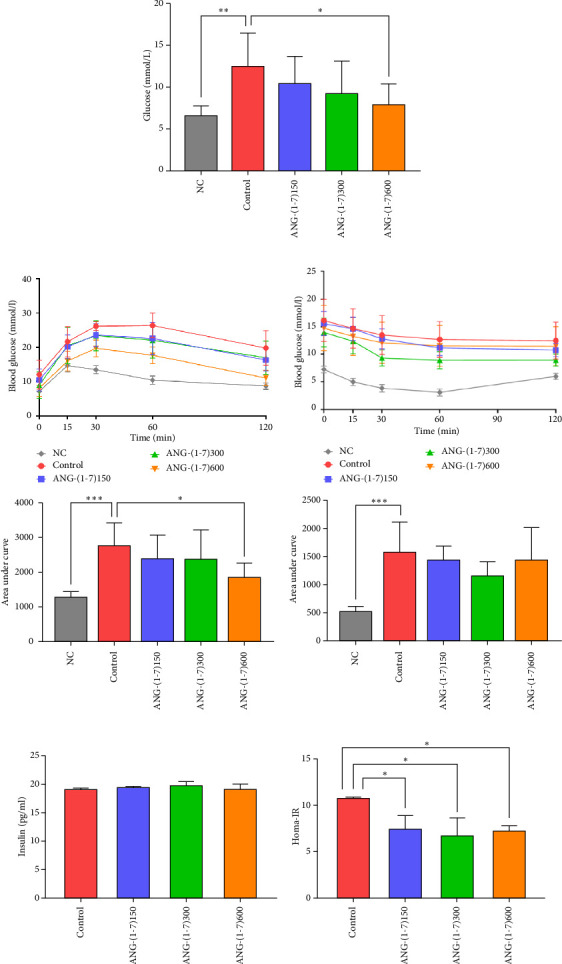
The effect of angiotensin (1–7) on islet function in db/db mice. (a) The fasting blood glucose of mice under different interventions. *n* = 7–11 for each group. (b) The intraperitoneal insulin tolerance test (IPITT) results and the area under the curve (AUC) of IPITT. *n* = 7–11 for each group. (c) The insulin tolerance test (ITT) results and the area under the curve (AUC) of ITT. *n* = 7–11 for each group. (d) Quantification of serum insulin in db/db mice under different interventions. *n* = 3 for each group. (e) The homeostasis model assessment of insulin resistance (Homa-IR). *n* = 3 for each group. Bars represent the mean ± SD. Compared to control: ⁣^∗^*p* < 0.05, ⁣^∗∗^*p* < 0.01, ⁣^∗∗∗^*p* < 0.001.

The average FBG levels in the NC and Ang-(1–7) 600 groups were 6.63 and 7.96 mmol/L, both significantly lower than the control group's level of 12.51 mmol/L (Figure 3(a)). Notably, the area under the curve (AUC) in the Ang-(1–7) 600 group was significantly reduced compared to the control group during IPITT, indicating improved glucose tolerance (Figure 3(b)). Results from the ITT showed no significant difference between the intervention groups and the control group (Figure 3(c)). Serum insulin levels did not differ significantly among the groups, while the Homeostasis model assessment of insulin resistance (Homa-IR, HOMA⁃IR = FPG × Fins/22.5) of the intervention group decreased significantly (Figures 3(d) and 3(e)).

### 3.4. Angiotensin (1–7) Increased the Number of Islets in db/db Mice

**Figure 4:**
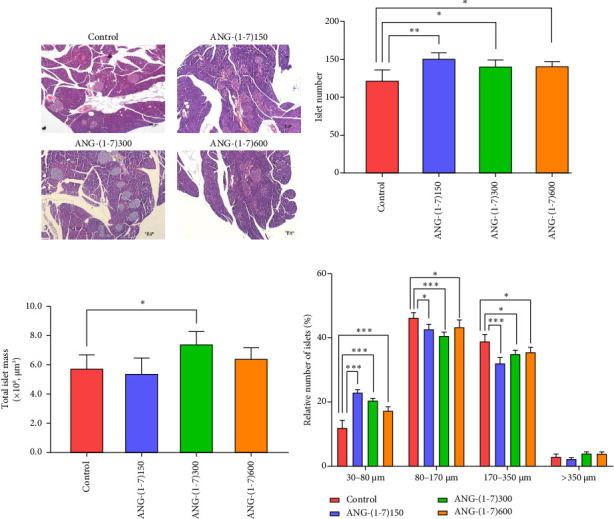
The effect of angiotensin on the morphology of pancreatic islets in db/db mice. (a) Representative image of pancreas tissues stained with HE from the control group, ANG-(1–7)150 group, ANG-(1–7)300 group, and ANG-(1–7)600 group. Original magnification ×100. (b) The counts of pancreatic islets under different interventions. (c) Quantification of total islet mass under different interventions. (d) The distribution of islet size in db/db mice with different interventions. Bars represent the mean ± SD. Compared to control: ⁣^∗^*p* < 0.05, ⁣^∗∗^*p* < 0.01, ⁣^∗∗∗^*p* < 0.001. *n* = 5 for each group.

To assess the quantity and size of islets, HE staining was conducted. Analysis revealed that the angiotensin (1–7) intervention group exhibited an increase in both the number and average area of islets (Figure 4(a)). Notably, the number of islets in the angiotensin (1–7) intervention group showed a significant increment compared to the control group (Figure 4(b)). Furthermore, the ANG-(1–7)300 group demonstrated an approximate 30% increase in total islet volume (Figure 4(c)). In order to gain further insights into the relationship between islet number and mass, we classified the islets based on their size. The angiotensin (1–7) intervention groups displayed an augmentation in the number of small islets (30–80 *μ*m), while the number of medium-sized islets (80–350 *μ*m) significantly decreased (Figure 4(d)).

### 3.5. Angiotensin (1–7) Increased the Beta-Cell Ratio and Beta-Cell Mass

**Figure 5:**
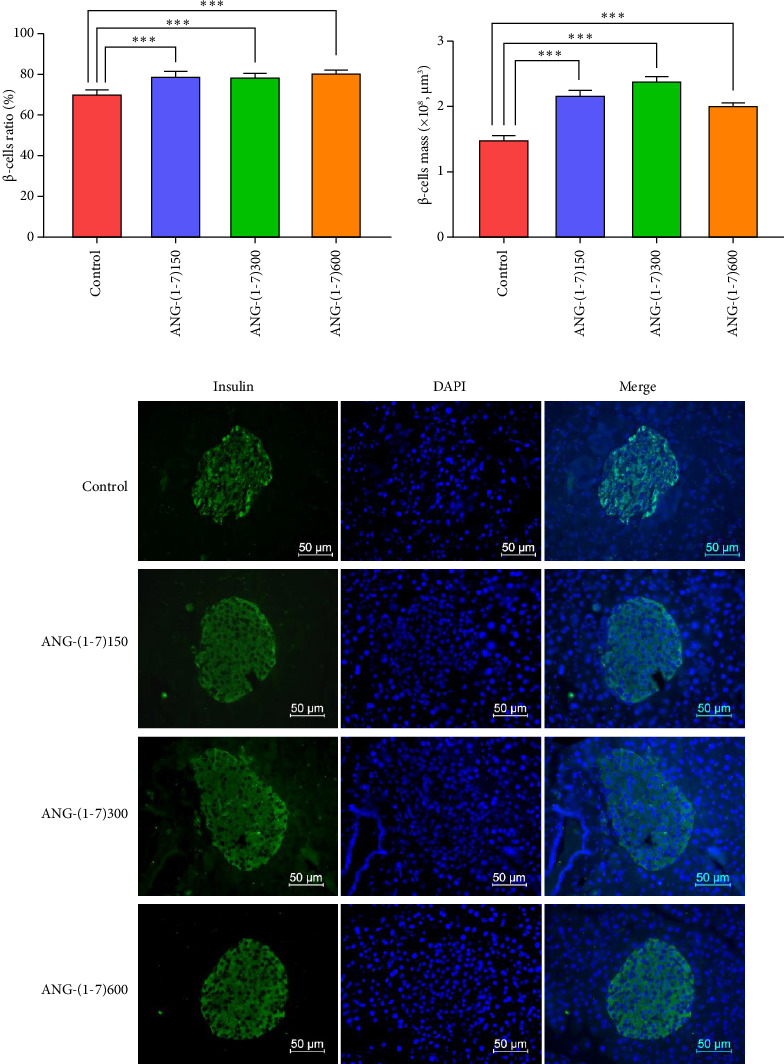
Angiotensin (1–7) increased *β*-cell mass in db/db mice. (a) Quantification of the *β*-cell ratio of islets in db/db mice; (b) histogram of *β*-cell mass; (c) immunofluorescence staining of insulin (green) was performed on the *β*-cells. Original magnification ×400. Each bar represents the mean ± SD. Compared to control: ⁣^∗^*p* < 0.05, ⁣^∗∗∗^*p* < 0.001. *n* = 5 for each group.

Immunofluorescence staining of pancreatic islets was performed to assess the *β*-cell ratio and *β*-cell mass. The results revealed that the proportions of *β* cells in the ANG-(1–7)150, ANG-(1–7)300, and ANG-(1–7)600 groups were 78.80%, 78.60%, and 80.60%, respectively, which were significantly higher than the 70.40% observed in the control group (Figures 5(a), 5(c)). Notably, the total mass of *β* cells also exhibited an increase in all angiotensin (1–7) intervention groups (Figure 5(b)).

### 3.6. Angiotensin (1–7) Elevated the Expression of PDX-1 and GCK in the Pancreas

**Figure 6:**
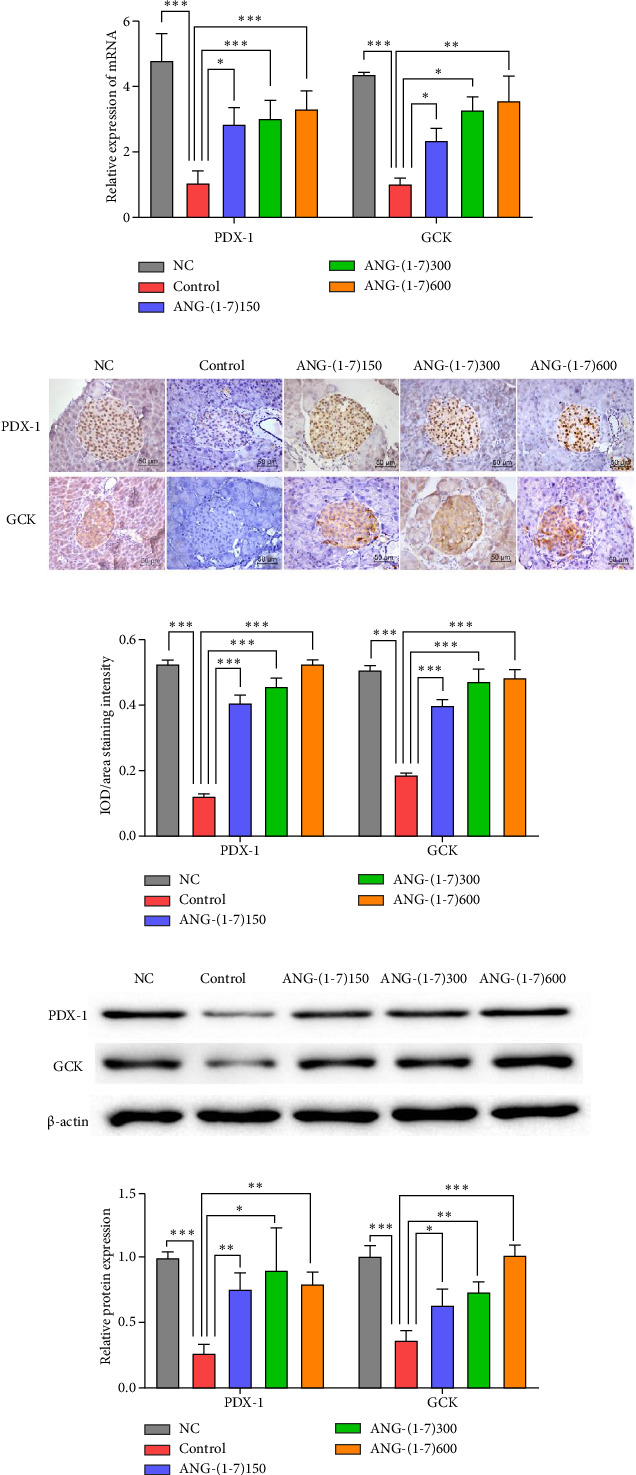
The expression of PDX-1 and GCK in db/db mice. (a) Relative expression of PDX-1 and GCK mRNA in the pancreas. *n* = 3 for each group. (b) Immunohistochemical staining of PDX-1, GCK in islet cells of mice. Original magnification ×400. *n* = 5 for each group. (c) Semiquantitative comparison of staining intensity of PDX-1 and GCK in islet cells of mice, which was determined by Image-Pro Plus. *n* = 5 for each group. (d) The expression of PDX-1 and GCK protein in the pancreas. (e) Semiquantification comparison of relative expression of PDX-1 and GCK, which was determined by Image-Pro Plus. *n* = 3 for each group. Bars represent the mean ± SD. Compared to control: ⁣^∗^*p* < 0.05, ⁣^∗∗^*p* < 0.01, ⁣^∗∗∗^*p* < 0.001.

To investigate the expression of PDX-1 and GCK, we assessed their mRNA and protein levels in the pancreas using RT-qPCR, IHC, and Western blotting. The results demonstrated a significant upregulation of both PDX-1 and GCK mRNA and protein expression in the angiotensin (1–7) intervention group compared to the control group (Figure 6). IHC revealed that PDX-1 was predominantly localized in the nucleus, while GCK was mainly expressed in the cytoplasm (Figure 6(b)). The staining intensity of PDX-1 and GCK in the islets of the intervention groups was significantly higher than that in the control group.

## 4. Discussion

Previous animal model studies have primarily focused on the beneficial effects of angiotensin (1–7) on the cardiovascular system. Administration methods such as intraperitoneal injection, gavage, or osmotic pump have been utilized, with intervention dosages ranging from 150 to 600 *μ*g/kg/d. To further investigate the metabolic effects of angiotensin (1–7), we conducted a study on glucose metabolism in db/db mice using three different doses of angiotensin (1–7). During the 8-week pharmacological intervention phase, there were no significant differences in food intake between the angiotensin (1–7) group and the control group (Figures 1(a)/1(b)). However, body weight was observed to be lower in the angiotensin (1–7) intervention group, particularly in the high-dose group (Figures 1(c)/1(d)). This weight loss may be attributed to two potential factors. Firstly, angiotensin (1–7) may enhance the basal metabolic rate, leading to increased energy expenditure and subsequent weight loss. In a mouse model of diet-induced obesity, angiotensin (1–7) intervention has been shown to elevate thermogenesis in subcutaneous white adipose tissue, induce brown adipocyte differentiation, and upregulate the expression of uncoupling protein-1 (UCP-1). These effects contribute to improved thermogenesis and amelioration of metabolic disorders [12, 13]. Secondly, recent studies have confirmed that the intervention of angiotensin (1–7) enhances insulin sensitivity and stimulates glucose uptake in adipocytes and the secretion of adiponectin by mice primary adipocytes, increases the expression of adiponectin in plasma and adipose tissue [14], and then increases fatty acid metabolism by activating AMPK [15–17], resulting in reduced abdominal fat content and weight loss. Recent evidence shows that angiotensin (1–7) is inversely associated with body weight, body mass index, leptin, and diastolic and systolic blood pressure [18].

In the angiotensin (1–7) intervention groups, varying degrees of decrease in TG were observed, while HDL and LDL levels showed an upward trend (Figures 2(a)/2(c)/2(d)). However, no significant differences were found in TC (Figure 2(b)). This lack of significant differences in some lipid metabolism indicators may be attributed to the inability of angiotensin to directly affect lipid metabolism. It is worth mentioning that angiotensin (1–7) treatment has been shown to improve lipid metabolism by increasing leptin levels [18]. However, it should be noted that the experimental mice used in this study are leptin receptor gene knockout models, which could potentially influence the results. The impact of this genetic modification on the relationship between angiotensin (1–7) intervention and lipid metabolism warrants further investigation. HE staining of liver tissues revealed a reduction in liver fat content and normal hepatocyte size and arrangement in the angiotensin (1–7) intervention group (Figure 2(e)). Previous studies have demonstrated that angiotensin (1–7) intervention can decrease total fat volume, serum TGs, and epididymal adipocytes, while improving glucose tolerance, insulin sensitivity, and adipogenic differentiation in fatty liver, obesity, steatosis, and muscle tissue [19]. These findings align with the observed decrease in TGs in our study. Interestingly, according to the PANDORA hypothesis reviewed by Petrov MS, fatty change of the pancreas may play an important role in the development of diabetes of the exocrine pancreas and exocrine pancreatic insufficiency [20]. It may increase the risk of diabetes by affecting pancreatic *β*-cell function and insulin secretion. More investigation on intra-pancreatic fat deposition is needed to verify this hypothesis.

A striking result of this study was that angiotensin (1–7) demonstrated the ability to improve glucose metabolism in db/db mice. Both the GTT and ITT showed a downward trend in the AUC, indicating improved glucose handling (Figures 3(b)/3(c)). Furthermore, the islet sensitivity index also exhibited varying degrees of improvement (Figure 3(e)). Insulin resistance, often caused by glucotoxicity and lipotoxicity, is a major contributor to impaired glucose metabolism [21, 22]. Furthermore, the angiotensin (1–7) intervention group exhibited smaller but more numerous islets compared to the control group. Specifically, the angiotensin (1–7) group showed an increased number of small islets (30–80 *μ*m) and a decrease in the number of large islets (170–350 *μ*m) (Figure 4(d)). Previous studies have demonstrated that drugs can influence the size of pancreatic islets. For instance, interventions with metformin and pioglitazone have been shown to increase islet size [23, 24]. Additionally, islet size may be associated with aging, as older animals tend to have larger islets with reduced mitotic activity [11]. We posit that these smaller islets represent a younger, newborn state, while the increase in islet size may lead to more severe internal ischemic hypoxic conditions. Thus, we consider the increase in small islets as a benign manifestation when compared to an overall increase in islet size.

Another important finding in this study was that the intervention with different doses of angiotensin resulted in an increased proportion and total mass of *β* cells (Figures 5(a)/5(b)), as well as the increase of insulin in *β* cells (Figure 5(c)). PDX-1 and GCK play pivotal roles as regulators in promoting insulin synthesis and secretion [25, 26]. PDX-1 is crucial in inducing *β*-cell differentiation, facilitating pancreatic development, and maintaining normal islet structure [27]. Studies have reported enhanced sensitivity to apoptotic factors in PDX-1 (±) islet cells, accompanied by decreased anti-apoptotic proteins Bcl-2 and Bcl-(XL) levels, and increased caspase-3 activity, ultimately leading to a reduction in the number of beta cells and islets through apoptosis [28]. On the other hand, GCK is primarily expressed in pancreatic *β* cells and hepatocytes and acts as a key enzyme in regulating glucose metabolism and insulin secretion. It serves as a glucose sensor, stimulating insulin secretion and being regulated by PPAR-*γ*, thereby playing a crucial role in *β*-cell reconstruction in patients with diabetes [29]. In this study, we assessed the expression of PDX-1 and GCK through RT-qPCR, IHC, and Western blot assays. The results consistently indicated significantly higher expression levels of PDX-1 and GCK in the angiotensin (1–7) intervention group compared to the control group (Figure 6). Based on these findings, we propose that angiotensin (1–7) improves pancreatic islet cell structure through the PDX-1/GCK pathway.

Research confirmed that the PDX-1 gene was transfected into insulin-secreting cells induced by rat BMSCs, and overexpression of PDX-1 has been shown to induce insulin gene expression and insulin secretion in a dose-dependent manner [30]. Additionally, GCK functions as a glucose sensor in pancreatic *β* cells. Its low glucose affinity allows for the modulation of enzymatic activity within the physiological range of glucose concentrations, thus playing a crucial role in regulating insulin secretion. Mutations in the GCK gene have been linked to hyperglycemia or hypoglycemia, with heterozygous and homozygous mutations resulting in maturity-onset diabetes of the young (MODY) and permanent neonatal diabetes (PNDM), respectively [31]. The binding of PDX-1 to the cis-acting element hupe3 in the promoter region of the GCK gene activates transcription and promotes glucose-dependent insulin release [32]. In our study, we observed an increase in the expression of PDX-1 and GCK in the pancreas (Figure 6). Within mature *β* cells, PDX-1 is known to transactivate the insulin gene along with other genes involved in glucose sensing and metabolism, such as GLUT-2 and GCK. Based on these findings, we hypothesize that angiotensin (1–7) improves glucose metabolism through its modulation of PDX-1 and GCK.

In conclusion, our study demonstrates that angiotensin (1–7) intervention significantly improves FBG levels and glucose tolerance in mice. It leads to a remarkable increase in the total volume and number of islets. Importantly, we observed significantly higher expression levels of PDX-1 and GCK in the islets of mice treated with angiotensin (1–7) compared to the control group. These findings highlight the crucial role of angiotensin (1–7) in improving glucose and lipid metabolism as well as enhancing islet function through the upregulation of PDX-1 and GCK genes in db/db mice islets. Angiotensin (1–7), as a novel member of the RAS, exerts its effects by binding to Mas receptors and counteracting the biological effects of Ang II. This mechanism ultimately leads to an improvement in insulin resistance. Therefore, further exploration of the ACE2-Ang-(1–7)-Mas receptor axis and its impact on metabolic syndrome, along with elucidating related mechanisms, could provide new therapeutic targets for the treatment of diabetes.

## 5. Limitations

While our study provides valuable insights, it is not without limitations. We focused on the role of PDX-1 and GCK in islet function improvement but did not examine other crucial genes involved in *β*-cell function, such as glucose transporter-2 and insulin itself. Additionally, the downstream signaling pathways of the Ang-(1–7)-Mas axis, which could offer deeper insights into the molecular mechanisms of angiotensin (1–7) action, were not investigated in this study. Future research should address these gaps to provide a comprehensive understanding of the effects of angiotensin (1–7) in diabetic models.

## Data Availability

Interested stakeholders may communicate with the corresponding author (Wencan Xu) to access de-identified datasets.

## Supporting Information

Graphical abstract was submitted as supporting file, which presented the overall framework, key findings, and highlights of this study.
