# Supplementary material for: Angiotensin (1–7) Improves Pancreatic Islet Function via Upregulating PDX-1 and GCK: A Dose-Dependent Study in Mice
Source: Int J Endocrinol. 2024 Dec 19;2024:1672096. doi: 10.1155/ije/1672096 (PMC11671625; doi:10.1155/ije/1672096)
Supplement: Supporting Information — Additional supporting information can be found online in the Supporting Information section. [file 1672096.f1.docx]

**Graphical Abstract**


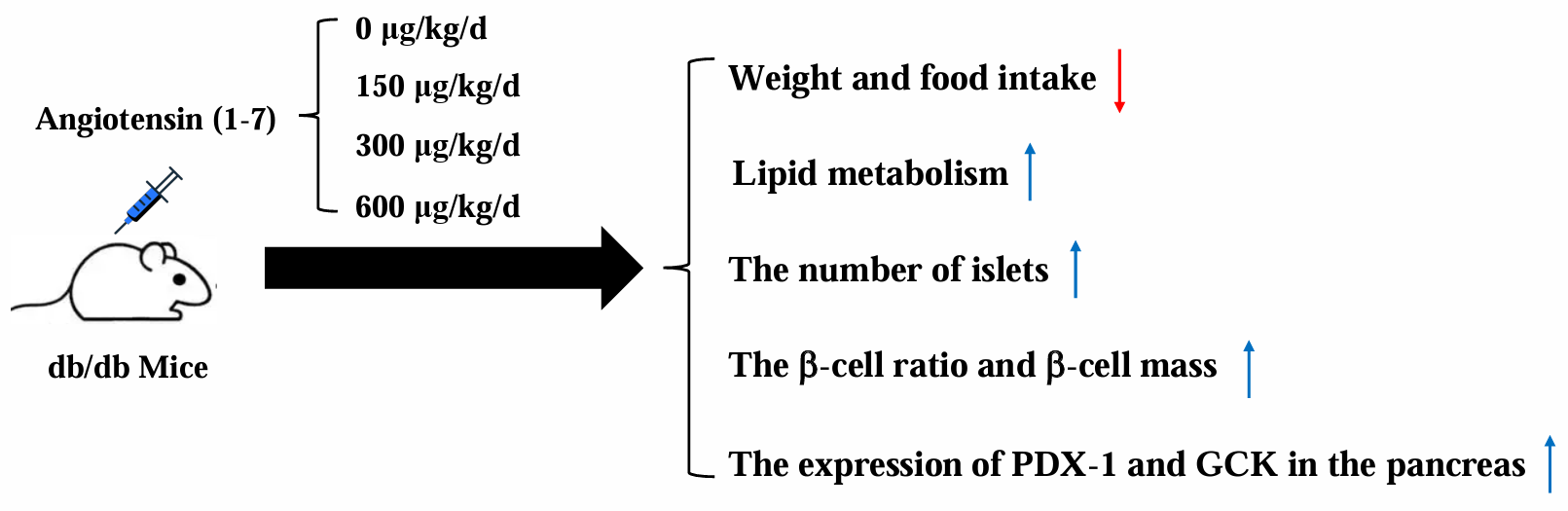


In this research, different doses of angiotensin (1-7) were injected to db/db mice daily for 8 weeks. Compared with the control group, we found a significant decrease in body weight and food intake in db/db mice receiving angiotensin (1-7) intervention. Besides, lipid metabolism of db/db mice was improved after intervention of angiotensin (1-7). Histological experiments showed that the number of islets, the β-cell ratio and β-cell mass were improved in db/db mice intervened with angiotensin (1-7). Extracting pancreatic tissue from db/db mice, we found that the expression of PDX-1 and GCK was significantly increased after intervention of angiotensin (1-7).
